# Evaluation of the Cellular Dissociation Grading, Based on Tumor Budding and Cell Nest Size, in Squamous Cell Carcinoma of the Penis

**DOI:** 10.3390/cancers14194949

**Published:** 2022-10-09

**Authors:** Hayel Derani, Anne-Sophie Becker, Oliver Hakenberg, Andreas Erbersdobler

**Affiliations:** 1 Institute of Pathology, University Medicine Rostock, 18055 Rostock, Germany; 2 Department of Urology, University Medicine Rostock, 18055 Rostock, Germany

**Keywords:** squamous cell carcinoma, penis, cellular dissociation grade, tumor budding, cell nest size, WHO grade, pN, lymph node metastases, lymphadenectomy, prognosis

## Abstract

**Simple Summary:**

The pathological status of regional lymph nodes (pN) is the most important predictor of survival in patients with invasive squamous cell carcinoma of the penis. However, staging strategies have limited prediction, which results in false-negative findings or unnecessary lymphadenectomies. Therefore, additional criteria should be identified to improve the prediction of lymph node involvement.

**Abstract:**

The “Cellular Dissociation Grade” (CDG) is based on tumor cell budding and cell nest size. Many studies have examined the CDG in squamous cell carcinomas of other organs such as the lungs, oral cavity, pharynx, larynx, cervix and esophagus. In this study, the CDG was examined in 109 cases of invasive penile squamous cell carcinoma that were treated at the University Medicine Rostock between 2014 and 2022. Furthermore, its correlation with the pathologic status of regional lymph nodes (pN) as the main prognostic factor was verified. Finally, cellular dissociation grading was compared with classic WHO grading. The results showed that pN in penile squamous cell carcinoma showed a highly significant association with the CDG and no statistically significant association with WHO grading. These results support the notion that cellular dissociation grading is an important prognostic factor for squamous cell carcinoma.

## 1. Introduction

The presence and number of lymph node metastases, as well as extranodal extent, are the most important predictors of survival in patients with invasive penile squamous cell carcinoma. However, staging strategies, whether noninvasive (clinical examination, imaging) or surgical (sentinel lymph node biopsy, staging lymphadenectomy), have been limited by a significant number of false-negative findings and/or high morbidity rates. Therefore, additional criteria should be identified to improve the prediction of lymph node involvement and reduce the number of unnecessary lymphadenectomies. The goal should be to identify the patients at the highest risk of inguinal lymph node metastases for inguinal lymphadenectomy, and to spare certain patients who do not benefit from lymphadenectomy [1]. Based on the current European guidelines, the indication for this procedure is not general, but is made based on the criteria “tumor infiltration, tumor grading and lymphovascular invasion” [2,3]. Due to the limitations of conventional grading systems regarding the prognosis of lymph node metastases, especially in squamous cell carcinoma of different organs, alternative grading systems have been sought.

The “Cellular Dissociation Grade” (CDG) is a novel histopathological grading system based on tumor cell budding (tumor budding) and cell nest size. Many studies have examined the CDG system in squamous cell carcinomas of other organs such as the lungs, head and neck, cervix and esophagus. The CDG system has been shown to outperform traditional grading systems in terms of prognosis [4,5,6,7,8]. A similar prognostic value for the CDG system was also found when evaluating preoperative biopsies or tumor tissue after neoadjuvant radiochemotherapy [9,10,11]. Tumor budding, a part of cellular dissociation grading, has been shown to be a prognostic factor in colorectal adenocarcinoma in several studies [12,13,14,15,16,17]. The aim of the present study was to evaluate the CDG in penile squamous cell carcinoma, especially with respect to the prognostic parameter “Lymph node metastasis”. 

## 2. Materials and Methods

### 2.1. Data Collection

This study is based on the data of 109 patients who were treated for the diagnosis of squamous cell carcinoma of the penis in the period from 2014 to 2022 at the Rostock University Medical Center. Approval for this study was obtained from the ethics committee of the Rostock University Medical Center under registration number A2020-0192.

The cohort consisted of 109 cases of invasive squamous cell carcinomas of the penis. The following data were collected: 

a- Patient age at diagnosis.

b- Pathological grade according to the WHO (WHO-G): On the basis of architectural disorder and cell atypia, the tumors were graded according to WHO grading into well-differentiated (WHO-G1), moderately differentiated (WHO-G2) and poorly differentiated (WHO-G3) tumors. Well-differentiated tumors show cytological features such as normal squamous epithelium, but with irregular nest structures. Moderately differentiated carcinomas show more nuclear pleomorphism, mitoses and cell atypia. Poorly differentiated tumors have a high rate of mitosis, including abnormal mitoses and little keratin formation [18,19].

c- Pathological status of TNM classification according to the WHO [19,20]. In 44 cases, no inguinal lymphadenectomy was performed, and consequently the pathological status of the lymph nodes was not available (pNx).

### 2.2. Microscopy

The hematoxylin-and-eosin-stained histological sections of the cohort and, where applicable, the sections of the special stains and immunohistochemistry were collected from the archive and re-evaluated. Further immunohistological examinations (CK5/6) were carried out, if necessary, to better assess the tumor dissociation in the areas with extensive peritumoral inflammation. 

The cases were graded according to the cellular dissociation grading system by three pathologists (H.D., A.-S.B., and A.E.). All observers examined the sections as they were blind to further clinical information and the grading results of the other pathologists. The high-power field (HPF) was typically evaluated at 400× magnification with a diameter of 0.55 mm and an area of 0.238 mm^2^ [21].

### 2.3. Cellular Dissociation Grade (CDG) 

The CDG is based on the criteria of “tumor budding” and “cell nest size”, according to the studies of Jesinghaus and Boxberg (Table 1) [4,5,6,7,8,9,10,11]. The HPF with the highest “budding activity” was chosen for the evaluation. In addition, the smallest cell nest size of the tumor cells in the available sections was sought.

1- Tumor budding (TB): A tumor bud was defined as a cell nest of less than 5 tumor cells. Tumors without budding activity received a score of (1). A score of (2) was assigned to tumors with low budding activity (<5 buds per HPF). The tumors with high budding activity (≥5 buds per HPF) received a score of (3).

2- Cell nest size (CNS): The tumors with large cell nests (>15 cells/nest) received a score of (1). Tumors with 5–15 cells/nest received a score of (2). Small nests (2–4 cells/nest) were given a score of (3). Single-cell invasion was scored (4).

**Figure 1:**
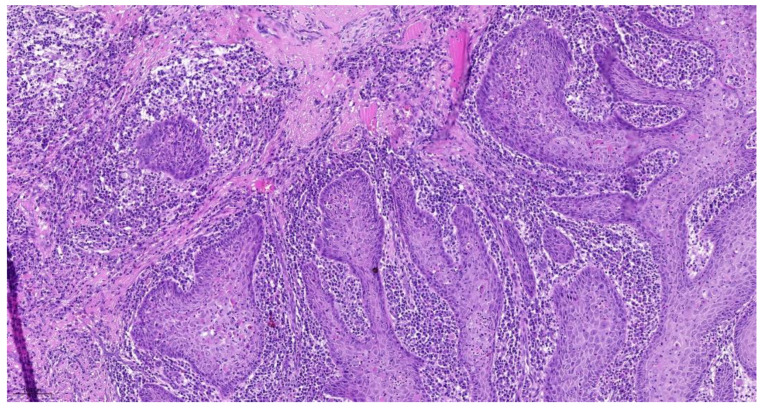
CNS(1) + TB(1) = CDG-1 _10.0×.

**Figure 2:**
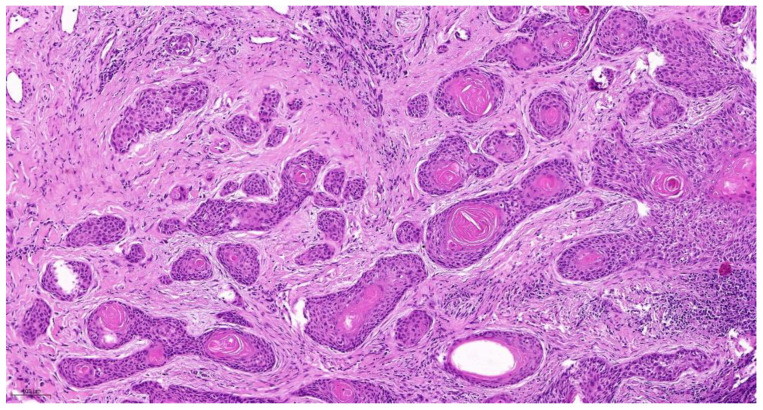
CNS(3) + TB(2) = CDG-2 _10.0×.

**Figure 3:**
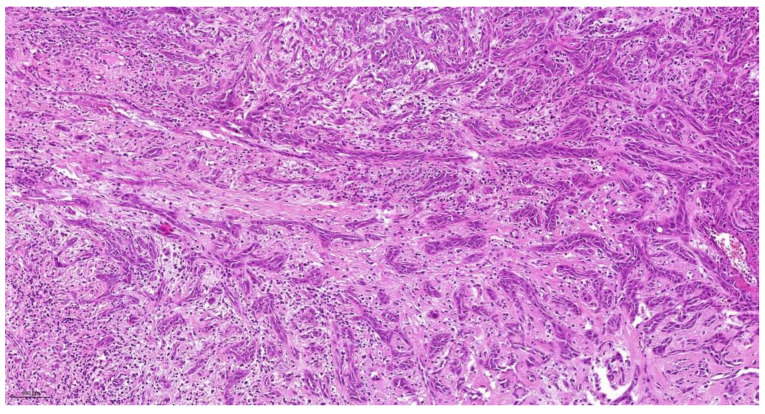
CNS(4) + TB(3) = CDG-3 _10.0×.

The cellular degree of dissociation (CDG) results from the sum of both variables (TB + CNS), which varies between 2 and 7 (Table 1). The well-differentiated tumors by CDG grading (CDG-1) had a sum ranging from 2 to 3 (Figure 1), moderately differentiated tumors (CDG-2) had a sum ranging from 4 to 5 (Figure 2), and the poorly differentiated carcinomas (CDG-3) had a total of 6 to 7 (Figure 3) [4,5,6,7,8,9,10,11].

### 2.4. Statistics

All data were entered into an Excel spreadsheet. Tables and charts were created using Microsoft Excel 2016 and IBM SPSS Statistics version 27. All statistical calculations were performed using the IBM SPSS Statistics Version 27 program. Age was treated as a metric variable. In contrast, all other parameters were treated as ordinal variables because they have a natural order without metric spacing. Simple frequencies were calculated statistically.

In the course of examining the relationship between “lymph node status (pN)” and following parameters: “WHO grade (WHO-G)”, “cellular dissociation grade (CDG)”, “tumor budding (TB)” and “cell nest size (CNS)”, a respective cross table with chi-square test as well as univariate analysis of the between-subjects effects were used. The results were rated as significant with a *p*-value < 0.05 and as highly significant with a *p*-value < 0.01. Moreover, the ROC curve analysis of WHO-G, CDG, TB and CNS in discriminating pN0/N1 status was created. The worst value of the area under the curve is 0.5 by definition.

## 3. Results

### 3.1. Distribution of Collective Characteristics

Patient age: Patient age at diagnosis ranged from 29 to 90 years with a median of 65. 

**Figure 4:**
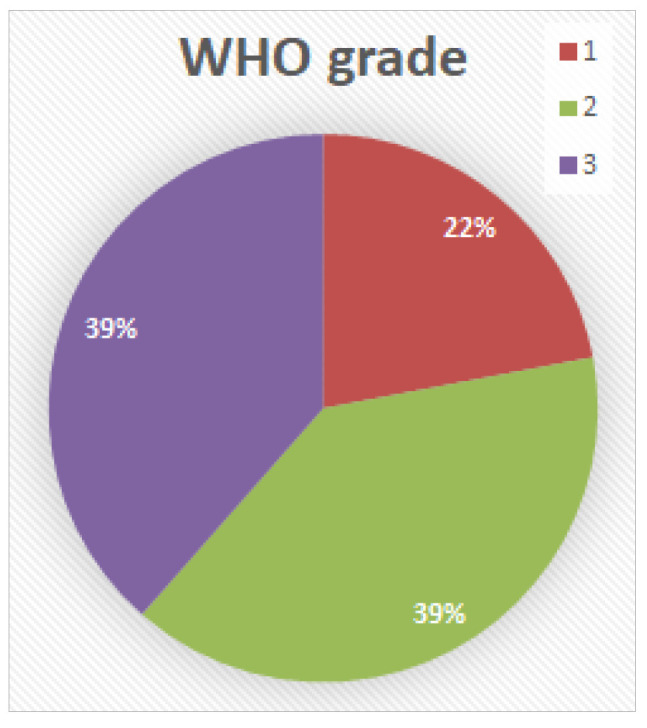
WHO grade distribution.

WHO grade (Figure 4): In 24 of a total of 109 cases (22%), the squamous cell carcinoma was assessed as WHO-G1, in 43 cases (39.5%) as WHO-G2, and in 42 cases (38.5%) as WHO-G3. 

**Figure 5:**
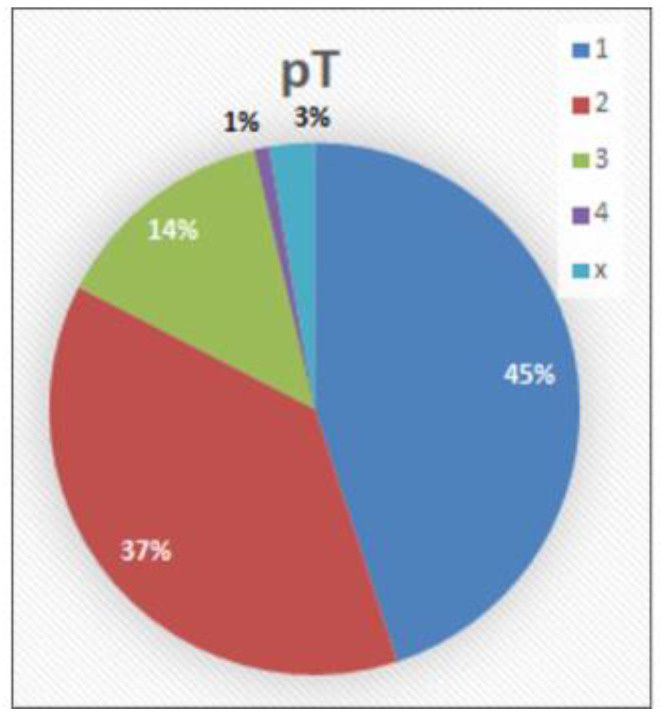
pT distribution.

pT category (Figure 5): The carcinoma infiltrated the subepithelial connective tissue (pT1) in 49 cases (45%), the corpus spongiosum (pT2) in 41 cases (37.6%), the corpus cavernosum (pT3) in 15 cases (13.8%), and other neighboring structures (pT4) in a single case (0.9%). In 3 cases (2.8%), pT could not be assessed because the tissue material was a biopsy without any other internal pathological findings.

**Figure 6:**
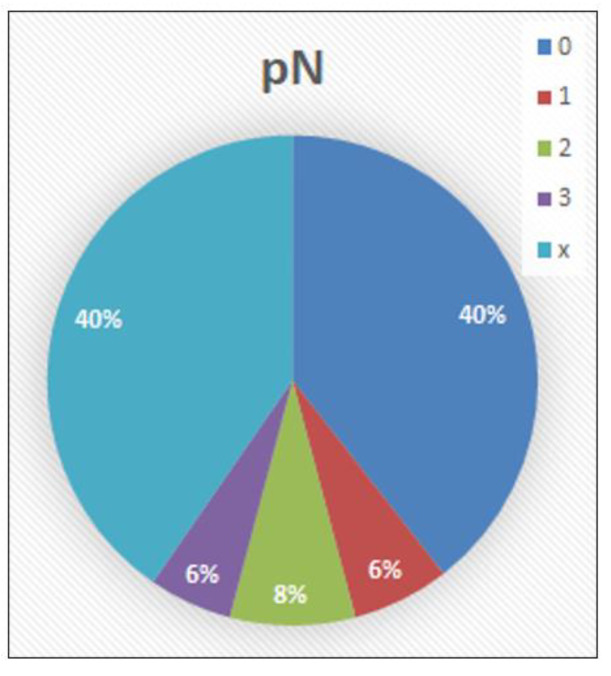
pN distribution.

pN category (Figure 6): The pathological lymph node status was known in 65 cases (59.6%). In the other 44 cases (40.3%), no lymphadenectomy was performed (pNx). In 43 cases (39.4%), the histological examination of the regional lymph nodes revealed no evidence of metastases (pN0). In seven cases (6.4%), metastases were found in one or two inguinal lymph nodes (pN1). In nine cases (8.3%), metastases were found in more than two unilateral inguinal or bilateral inguinal lymph nodes (pN2), and in six cases (5.5%), metastases were found in pelvic lymph nodes (unilateral or bilateral) or extranodal extension was detected (pN3). 

### 3.2. Comparison of Grading Systems

H.D. was the principal investigator for CDG. All diagrams of the correlations between CDG and WHO-G, and between CDG and pN were performed with the data obtained by the principal investigator.

#### 3.2.1. Distribution of Grading Systems

**Figure 7:**
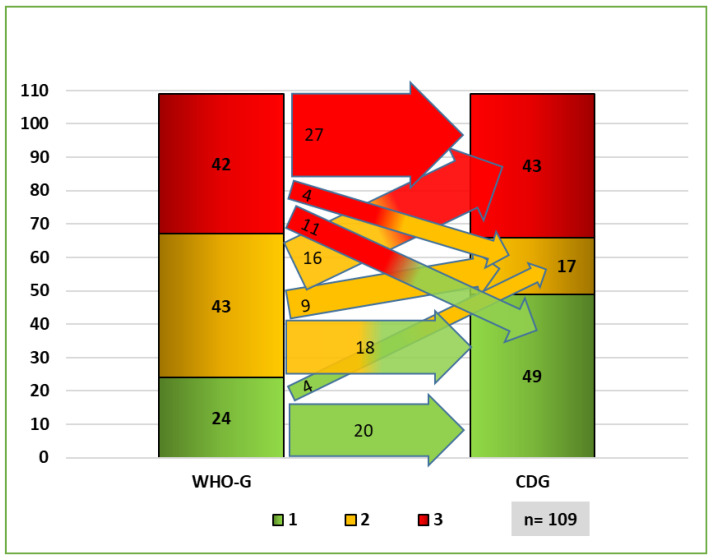
Flowchart of distribution of both grading systems.

A prominent part (13.7%) of the cases with poor differentiation according to the WHO (WHO-G3) were assessed as not poorly differentiated according to CDG grading (CDG-1/2). Moreover, a prominent part (14.6%) of the not poorly differentiated tumors according to the WHO (WHO-G1/2) were assessed as poorly differentiated according to CDG grading (CDG-3) (Figure 7). This difference is relevant for differentiating between pT1a and pT1b in the pT stage, as pT1a tumors are not poorly differentiated/undifferentiated, and pT1b tumors are poorly differentiated/undifferentiated [18,19,20].

**Figure 8:**
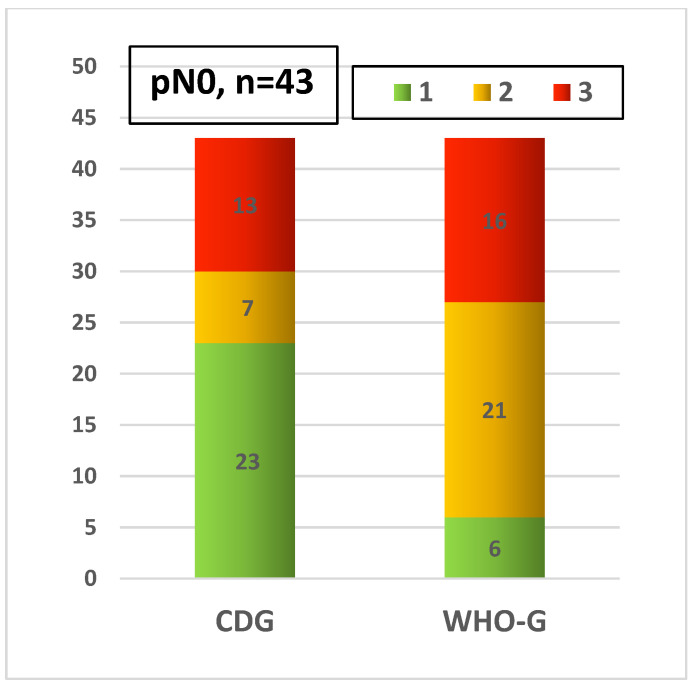
Distribution of both grading systems with pN0 cases.

**Figure 9:**
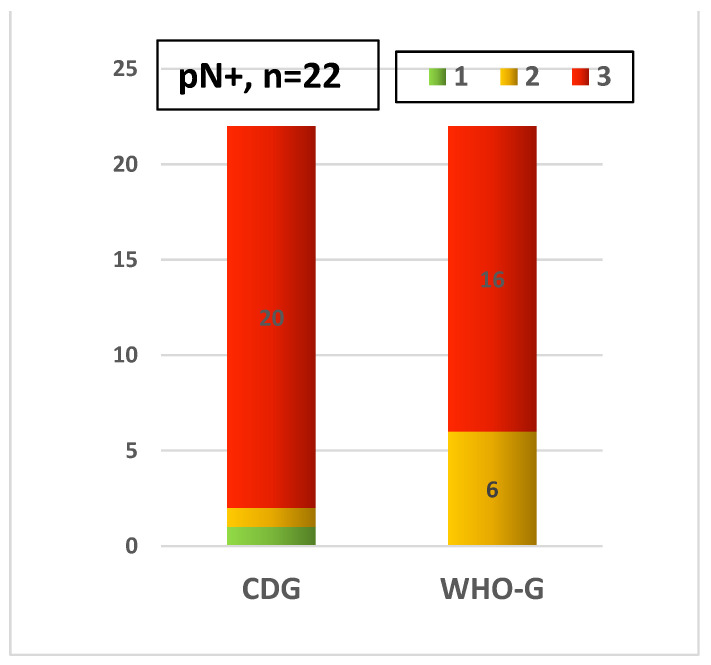
Distribution of both grading systems with pN+ cases.

The comparison of the grading systems with regard to the pN status shows significant differences too. WHO-G1 was less commonly (13.9%) associated with pN0 compared to CDG-1 (53.4%). On the other hand, WHO-G2 was more frequently present in pN0 cases compared to CDG-2 (Figure 8). Cases with pN+ also showed differences in the distribution between WHO-G and CDG (Figure 9).

#### 3.2.2. Prognostic Validity of Grading Systems for Lymph Node Status (pN)

Two-tailed asymptomatic significance on Pearson’s chi-squared test between pN and WHO-G was *p* = 0.112 > 0.05. Almost all values of chi-squared between pN and (CDG, TB, CNS), respectively, were *p* < 0.05 and sometimes *p* < 0.01 with all observers (Table 2). Therefore, the pathologic status of regional lymph nodes (pN) in penile squamous cell carcinoma showed a significant or highly significant association with cellular dissociation grading (CDG) and its components (tumor budding TB, cell nest size CNS), but no statistically significant association with the WHO grading.

In the univariate analysis, WHO-G showed significant between-subjects effects with pN. However, CDG, TB, CNS showed significant or highly significant between-subjects effects with pN with all observers.

**Figure 10:**
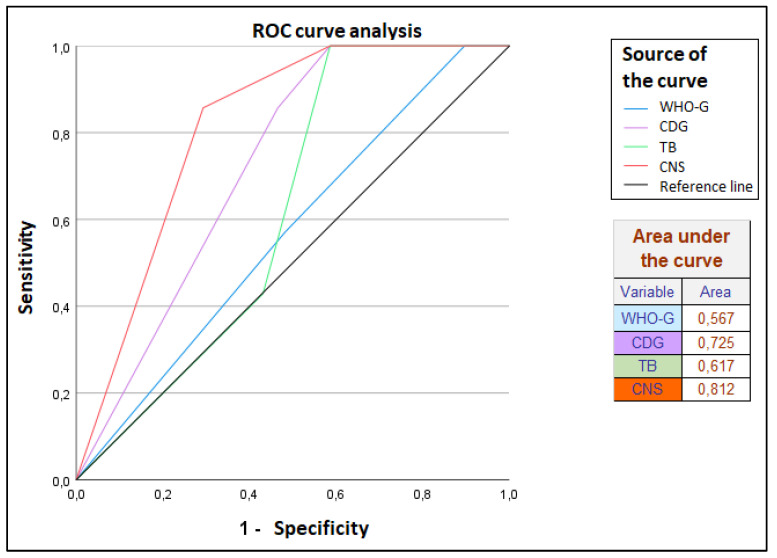
ROC curve regarding prognosis for pN.

In the ROC curve analysis of CDG and WHO grading in discriminating pN0/N1 status, the area under the curve of CDG, TB and CNS is significantly higher than 0.5 compared to the WHO-G, so the hit rate of CDG, TB and CNS with regard to pN is significantly better than that of WHO-G (Figure 10).

### 3.3. Interobserver Concordance 

The kappa coefficient of the interobserver concordance was moderate for CDG according to Landis and Koch [22] (κ = 0.512) (Table 3). We did not test the intraobserver concordance (test–retest concordance).

### 3.4. CDG Spectrum of the Three Pathologists 

**Figure 11:**
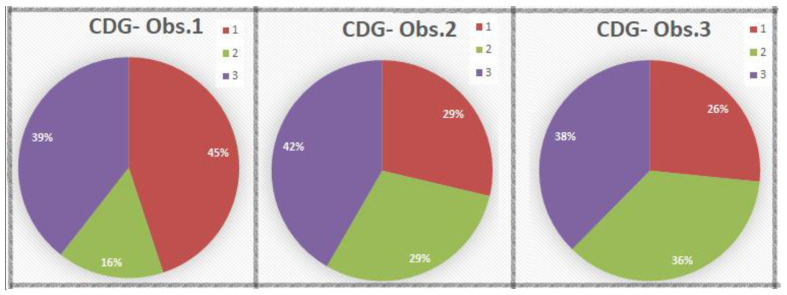
CDG distribution for all observers.

CDG-1 was assigned in a range of 29–49 cases, 26.6–45%, mean 33.4%, CDG-2 in a range of 17–39 cases, 15.6–35.8%, mean 27%, and CDG-3 in a range of 41–45 cases, 37.6–41.3%, mean 39.5% (Figure 11). 

## 4. Discussion

### 4.1. Significance and Problems of the WHO Grading in Penile Carcinoma 

In penile carcinoma, the grading plays an important role because it is part of the T classification and differentiates between pT1a and pT1b. As a result, it may be a factor in therapy planning. Despite this, the WHO does not prescribe a specific or precise grading system for penile carcinoma [23].

The differentiation of squamous cells varies greatly from the base to the surface in both normal squamous epithelium and squamous cell carcinoma (SCC). This phenomenon leads to heterogeneous patterns of differentiation and hampers grading in SCC. Grading is provided based on the worst observed grading pattern [18,19,23].

The presence of palpable inguinal lymph nodes (cN+) does not necessarily indicate the presence of lymph node metastases (pN+). The enlargement of the lymph nodes can have other reasons, e.g., age-related degenerative or inflammatory reasons. On the other hand, cN0 status does not necessarily indicate pN0 status. In a study with 71 cases of penis carcinoma, concordance (pN+/cN+) was only 61.5%, and concordance (pN0/cN0) was 62.2% [1]. 

The concordance between clinical and pathological staging was moderate in the other organs too. In 392 cases of oral squamous cell carcinoma, concordance between clinical and histopathologic staging was observed only in 60% of cases [24]. In 501 cases of head and neck squamous cell carcinoma, concordance between clinical and pathological staging was 52.2% for T category, 53.5% for N category and 54.9% for the overall stage [25].

### 4.2. Cellular Dissociation Grading (CDG) in International Studies

The CDG system has been investigated in several organs such as lung [26], oral cavity [5], pharynx and larynx [6], esophagus [9,10,11] and cervix [8]. The CDG grading in these studies was significantly correlated with overall, disease-specific and disease-free survival, and was found to be a strong age-, stage-, and sex-independent prognostic factor for survival. These results support the notion that a grading system based on cell nest size and tumor budding may be useful in squamous cell carcinoma, and appear to capture differences in the underlying biology of squamous cell carcinoma that are responsible for aggressiveness of the tumor. This could be helpful in deciding on treatment methods such as the extent of surgical resection, adjuvant and neoadjuvant chemotherapy [9], or expanded lymphadenectomy.

Tumor budding has similar prognostic significance to CDG in the lung [27,28,29,30], oral cavity [31,32], and pharynx and larynx [33,34] and is a recognized diagnostic criterion in adenocarcinoma of the colon [12,13,16,17,35].

### 4.3. Evaluation of Cellular Dissociation Grading (CDG) in Penile Carcinoma

In our study, we defined lymph node status as the most important prognostic factor. Every tumor grading should show a proportional assignment with this prognostic factor in order to gain prognostic relevance. The lowest grading score should indicate no lymph node metastases (pN0), and the highest grading score should be associated with lymph node metastases (pN+).

The results of our study showed that the prognostic value of the CDG system as a predictor for the lymph node metastases was significantly higher than that of the WHO grading system. The lower grades of the CDG system were more predictive of pN0 status than the low WHO grades, and the higher grades of the CDG system were more strongly associated with regional lymph node metastases (pN+).

The pathologic status of regional lymph nodes (pN) in penile squamous cell carcinoma showed a highly significant association with Cellular Dissociation Grading (CDG), and no statistically significant association with WHO grading (Table 2). These results are consistent with the international studies on other tumor entities (head and neck, esophagus, lung, cervix and colon) and support the notion that cellular dissociation grading is an important prognostic factor for squamous cell carcinoma.

### 4.4. Limitations and Problems of CDG Grading

In the case of dense peri-/intratumoral inflammation, immunohistochemistry with cytokeratin staining can help identify tumor buds [15]. An important problem is determining a valid cut-off point for tumor budding. For example, a cut-off value of 10 buds could be used to classify tumor budding into a low (≤10 buds/HPF) and a high grade (>10 buds/HPF) in colorectal cancer [36]. Another study considered a cut-off value of (25 buds/0.785 mm^2^ field of view) to be reasonable (equivalent to 7–8 buds/HPF) [36]. Ueno et al. graded tumor budding differently, namely as I, II, III, and IV, according to the number of budding units respectively 0–4, 5–9, 10–19, and >20 per HPF [37]. Tumor budding was consistently demonstrated as prognostically relevant by several groups with different assessment methods (after selecting the maximum budding site at low magnification) [29]: (a) assessment of budding in 1 HPF [38]; (b) assessment of budding in 10 HPFs, then selecting the maximum score in 1 HPF [26,28]; and (c) evaluating the budding in 10 HPFs, then calculating the average score [26,28]. In colorectal adenocarcinoma, tumor budding is divided into peritumoral budding (PTB, tumor buds at the tumor front) and intratumoral budding (ITB, tumor buds in the tumor center) [14]. In this study, we only examined peritumoral tumor budding in penile carcinomas.

### 4.5. Interobserver Variation

International studies show that the interobserver concordance was usually moderate in many established grading systems of various cancers. For example, WHO grading in cutaneous squamous cell carcinoma had a moderate overall concordance when 131 cases were graded by three dermatopathologists (κ = 0.56–0.60), with the lowest agreement in moderately differentiated tumors [39]. Moreover, the Gleason score of prostatic carcinoma had a barely moderate agreement (κ = 0.435) when assessed on 38 prostate biopsies by 41 pathologists [40].

In penile squamous cell carcinoma, the concordance of grading systems was also poor to moderate in many studies. The WHO grading of penile squamous cell carcinoma showed poor to moderate concordance between seven pathologists in 207 cases (κ = 0.23, range: 0.07–0.55) [41]. The College of American Pathologists (CAP) grading system [42] also showed overall low interobserver reproducibility (κ = 0.34, range 0.02–0.67) between 12 experienced pathologists when 90 penile carcinoma cases were graded according to this grading system [43].

The concordance of CDG grading in our study (κ = 0.512, range: 0.484–0.565) is better than the concordance of WHO grading and CAP grading in the reported studies, even without experience with this new grading system.

In a study about CDG in squamous cell carcinoma of the oral cavity, the interobserver agreement was moderate to almost perfect (κ = 0.55–0.97) with three pathologists. The almost perfect concordance was between the two pathologists who proposed this new CDG grading. However, the concordance improved after training the third observer (κ = 0.84–0.87) [4].

## 5. Conclusions

In our study, cellular dissociation grading has proven to be a factor for the prediction of lymph node metastases in squamous cell carcinoma of the penis. It is recommended that a grading system that includes cellular dissociation characteristics should be integrated in future recommendations by the WHO atlas for squamous cell carcinoma of the penis as a grading tool to improve prognosis. 

Further exploration of the relationship between cellular dissociation with specific tumor–host interactions and molecular events should be addressed in future studies. 

## Figures and Tables

**Table 1:** Cellular dissociation grading [4,5,6,7,8,9,10,11].

Histological Feature	Classification	Score & Grade
Tumor Budding * (TB)	**No tumor budding**	Score 1
	1–5 tumor buds/HPF	Score 2
	>5 tumor buds/HPF	Score 3
**Cell Nest Size (CNS)**	>15 cells per nest 5–15 cells per nest 2–4 cells per nest	Score 1 Score 2 Score 3
	single cell invasion	Score 4
**Cellular Dissociation Grade (CDG)**	Sum TB + CNS = 2–3 Sum TB + CNS = 4–5	CDG-1 CDG-2
	Sum TB + CNS = 6–7	CDG-3

* Tumor bud: Tumor cell nest with <5 cells.

**Table 2:** Relationship with pN.

Statistical Test	WHO-G	CDG	TB	CNS
Asymptotic Significance-Pearson Chi-Square	0.112	<0.001–0.033	<0.001–0.057	0.006–0.029
Between-subjects effects-univariate analysis	0.018	<0.001–0.010	<0.001–0.012	0.004–0.041

**Table 3:** Interobserver concordance for CDG evaluation.

Statistical Test	Cohens Kappa			Fleiss, Kappa Obs.1,2,3
	Obs.1 vs. Obs.2	Obs.1 vs. Obs.3	Obs.2 vs. Obs.3	
Value	0.495	0.484	0.565	0.512

## Data Availability

Data available on request due to privacy and ethical restrictions. Permissions of the corresponding author and Institute of Pathology at University Medicine Rostock are necessary. The data are not publicly available.
